# The Physiological Role of Irisin in the Regulation of Muscle Glucose Homeostasis

**DOI:** 10.3390/endocrines2030025

**Published:** 2021-08-13

**Authors:** Naohiro Yano, Yu Tina Zhao, Ting C. Zhao

**Affiliations:** 1 Roger Williams Medical Center, Department of Surgery, Boston University School of Medicine, Boston, MA 02118, USA; 2 Department of Plastic Surgery, Rhode Island Hospital, Brown University, Providence, RI 02912, USA; 3 Department of Imaging Sciences, University of Rochester Medical Center, Rochester, NY 14642, USA

**Keywords:** irisin, myokine, glucose homeostasis, type 2 diabetes mellitus, metabolic disorders

## Abstract

Irisin is a myokine that primarily targets adipose tissue, where it increases energy expenditure and contributes to the beneficial effects of exercise through the browning of white adipose tissue. As our knowledge has deepened in recent years, muscle has been found to be a major target organ for irisin as well. Several studies have attempted to characterize the role of irisin in muscle to improve glucose metabolism through mechanisms such as reducing insulin resistance. Although they are very intriguing reports, some contradictory results make it difficult to grasp the whole picture of the action of irisin on muscle. In this review, we attempted to organize the current knowledge of the role of irisin in muscle glucose metabolism. We discussed the direct effects of irisin on glucose metabolism in three types of muscle, that is, skeletal muscle, smooth muscle, and the myocardium. We also describe irisin’s effects on mitochondria and its interactions with other hormones. Furthermore, to consider the relationship between the irisin-induced improvement of glucose metabolism in muscle and systemic disorders of glucose metabolism, we reviewed the results from animal interventional studies and human clinical studies.

## 1. Introduction

Insulin resistance and abnormal insulin secretion are thought to be the major mechanisms of type 2 diabetes (T2DM) onset. Although there is a debate about the fundamental cause of T2DM, in general, insulin resistance is thought to precede its deficiency in the early stages of onset, and hyperglycemia develops when the relative lack of insulin exceeds the threshold. T2DM can be said to be a disease that includes various pathological conditions caused by hyperglycemia. It has been believed that sedentary behavior, commonly seen in subjects with T2DM, is associated with many deleterious health outcomes. Obesity, because of the associated sedentary behavior, is one of the most important modifiable risk factors for the prevention of T2DM. Accordingly, preventing TDM development and treating its associated consequences should focus on lifestyle modifications to eliminate a lack of exercise [1]. It is widely known that regular exercise has benefits for the treatment of patients with T2DM, such as improved bodyweight control, better blood glucose levels, greater regulated blood pressure control, and the onset of fewer complications [2,3]. Various prescriptions for exercise therapy are being tested, and some particular types of exercise, such as aerobic and resistance training, have been shown to be effective for the treatment of T2DM [4–6].

Muscle falls into three distinctly different types, as follows: myocardium, skeletal muscle, and smooth muscle. More than half of a body’s weight is made up of muscle, that is, muscle is the largest organ of the body. Muscle is also known as the largest site of insulin-stimulated glycogen synthesis for glucose storage. In addition, it has recently come to be recognized as a secretory organ capable of releasing various myokines [7]. Myokines regulate multi-organ metabolism, angiogenesis and growth through autocrine, paracrine and endocrine signaling [8]. Some of the myokines are induced by exercise, and exercise-induced myokines can have some beneficial biological effects, for example, anti-inflammatory effects in both acute inflammation and in chronic low-grade inflammation [9]. Gene expression in muscle and serum levels of myokines show unique patterns of change immediately after the start of exercise, suggesting that the exercise-induced release of myokines may play an important role in coordinating metabolism, leading to a beneficial effect on T2DM treatment [10]. The impact of exercise on myokine function is not yet fully understood. However, it has been reported that exercise induces crosstalk between muscle and adipose tissue via myokine [8], induces the interaction between myokine and other cytokines [11], and controls systemic inflammatory response [11].

The myokine’s secretome contains many cytokines that act on various tissues, such as adipose tissue, liver, pancreas, and brain [12–15]. Among them, irisin is a novel myokine produced by the release of the proteolytically cleaved extracellular portion of the fibronectin type III domain-containing protein 5 (FNDC5) [16]. Irisin is secreted in response to exercise and increases energy expenditure by promoting the browning of white adipose tissues (WAT) [17–19]. In mice fed a high-fat diet (HFD), the overexpression of *FNDC5* increased the serum levels of irisin, slightly reduced the weight, and, most prominently, improved hyperglycemia and hyperinsulinemia, suggesting an improvement in the insulin resistance of the mice [16]. Skeletal muscle also communicates with the pancreatic islet through irisin, regulating insulin secretion [20]. Thus, irisin has attracted a great deal of attention as a therapeutic target for metabolic diseases, including obesity, dyslipidemia, T2DM, and arterial hypertension. Based on these findings of *FNDC5* in metabolic regulation with the exercise-induced nature of irisin, and the possibility that muscle itself can be irisin’s target organ, researchers have started to look at the role of irisin in exercise-induced effects on muscle glucose metabolism [16,21,22]. The aim of this review is to highlight the emerging knowledge about irisin in glucose homeostasis in three types of muscles in vitro and in vivo under metabolic stresses, such as high-lipid/hyperlipidemia, and high-glucose/hyperglycemia.

## 2. Synthesis and Secretion of Irisin

**Figure 1.**
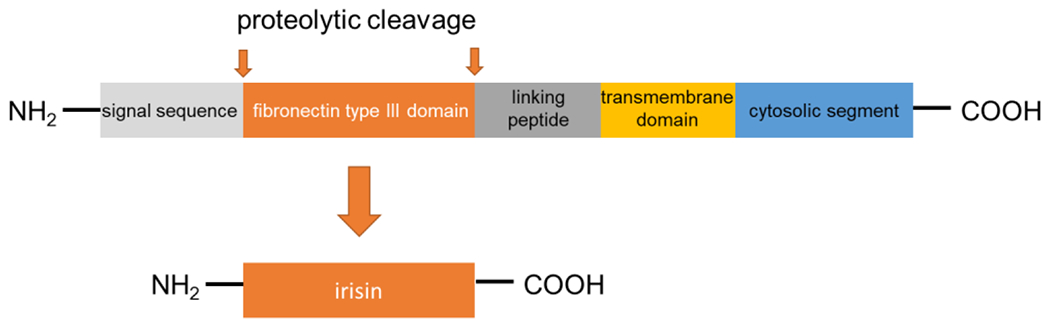
FNDC5 structure and formation of irisin.

Irisin was first described in 2012 as a myokine of transgenic mice overexpressing *Ppargc1a* (peroxisome proliferator-activated receptor gamma coactivator 1α; PGC1α), a transcription cofactor that plays a pivotal role in the regulation of energy metabolism [16]. PGC1α stimulates the expression of *FNDC5* to increase the synthesis of the membrane-bound FNDC5. FNDC5 is a 209-residue protein with an N-terminal 29-residue signal sequence, followed by a putative fibronectin III (FNIII) domain, a linking peptide, a transmembrane domain, and a 39-residue cytoplasmic segment (Figure 1). Proteolytically cleaved protein is modified with glycosylation and dimerization, and then the segment is released into circulation as irisin, which consists of 112 amino acids [23–25]. The 112-amino acid sequence is identical in humans and mice [16,26]. Previous research has revealed preliminary evidence that irisin is not only expressed in mammalian muscular tissues, such as cardiac muscle, skeletal muscle, and smooth muscle (tongue, rectum, etc.) but also in the pancreas, liver, and adipose tissue, which has important functions in systemic glucose metabolism regulation [27–30]. Therefore, it can be said that irisin belongs to the group of regulatory molecules, such as adipocytokines/adipokines [30–32]. Irisin induces the expression of uncoupling protein 1 (UCP1) and then increases energy expenditure in WAT with adipocyte browning [33]. Furthermore, irisin is expected to show protective effects in the pathogenesis of harmful complications of obesity, such as dyslipidemia, T2DM, and arterial hypertension [34–36]. With these findings, as mentioned above, irisin has attracted substantial interest as a novel remedy for these metabolic disorders. Of note, palmitate (PA), or high ambient glucose, inhibited the expression of *FNDC5* by human primary muscle cells in vitro [37]. However, *FNDC5* expression is generally higher in the muscle cells of individuals with T2DM than in those who are non-diabetic [37]. On the other hand, short-term (4 h) exposure of myotube to PA could induce irisin secretion without affecting FNDC5 [20]. Accordingly, HFD is able to acutely increase irisin serum concentration [20] These findings suggest that additional unknown factors are engaged in the lipid/glucose-mediated regulation of *FNDC5* expression. Future research is expected to disclose factors involved in the mechanism.

## 3. Muscle Glucose Homeostasis in Patients with Metabolic Diseases: A Role for Irisin

Metabolic diseases, such as T2DM, are diseases with rapidly increasing incidence that carry long-term harmful complications and cause premature death. The measurement factors in the pathogenesis of T2DM are insulin resistance, a deteriorated insulin secretory capacity, and a genetic background associated with excess energy intake and physical inactivity. Physical exercise, which directly protects muscle glucose metabolism and attenuates insulin resistance [38–40], may restore the impaired insulin secretory capacity [41] and rebuild glycemic control [42]. As mentioned above, irisin is induced by physical exercise, and given the biological activities of irisin, it is reasonable to presume that irisin is involved in the protective effects of physical training on muscular glucose metabolism.

**Figure 2.**
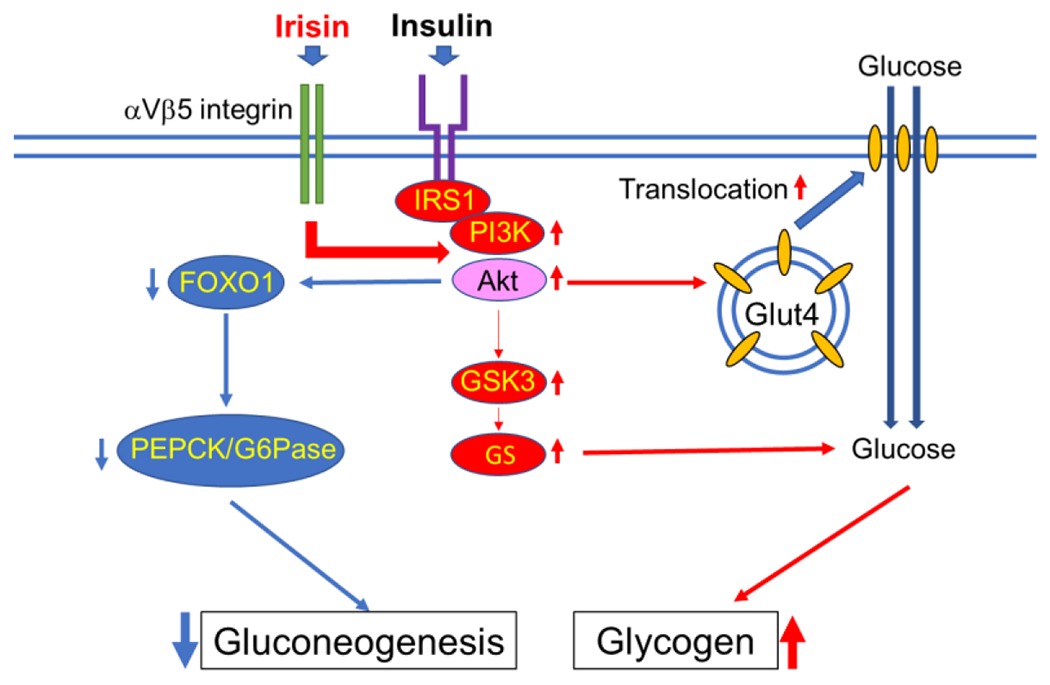
Irisin augments insulin-induced phosphatidylinositol 3-kinase (PI3K)/Akt signaling activity. The activated Akt promotes glucose transporter type 4 (GLUT4) translocation to the membrane, which leads to the increase of glucose inflow into the cell. For glycogen synthesis, the activated Akt inhibits GSK3 activity and subsequently activates glycogen synthase (GS) to enhance glycogen synthesis. Conversely, activated Akt inhibits forkhead box transcription factor O1 (FOXO1) and downregulates the gene expressions of phosphoenolpyruvate carboxykinase (PEPCK) and glucose-6-phosphatase (G6Pase), which leads to a decrease in gluconeogenesis. IRS: insulin receptor substrate. Thin red arrow indicates promotion, thin blue arrow indicates suppression.

Skeletal muscle plays a well-studied role in regulating glucose homeostasis, and skeletal muscle insulin resistance plays a pivotal role in the pathogenesis of T2DM [43]. By accounting for approximately 50% of the mass of the whole body, muscles make up a large part of the body capacity of glycogen storage. Under resting conditions, about 80% of blood glucose is metabolized by brain and peripheral tissues in an insulin-independent manner. However, after insulin stimulation, skeletal muscle accounts for almost 80% of glucose utilization [44]. Glycogen is the storage form of carbohydrates in mammals. In humans, the majority of glycogen is stored in skeletal muscle and the liver to a lesser extent. Glycogen storage in skeletal muscle is limited by feedback-mediated inhibition of glycogen synthase (GS), which prevents excess accumulation of glycogen. De novo lipid synthesis can take the place of glycogenesis when glycogen stores are filled [45], and this accelerated lipid synthesis will lead to ectopic fat accumulation and eventual insulin resistance [46]. Irisin improves glucose homeostasis by increasing glycogenesis via phosphatidylinositol 3-kinase (PI3K)/Akt/glycogen synthase kinase-3 (GSK-mediated glycogen synthase (GS) activation, while reducing gluconeogenesis via the downregulation of PI3K/Akt/forkhead box transcription factor O1 (FOXO1)-mediated phosphoenolpyruvate carboxykinase (PEPCK) and glucose-6-phosphatase (G6Pase) (Figure 2) [47]. The portion of the other types of muscle, that is, smooth muscle and myocardium, is far smaller than that of skeletal muscle. However, the glucose metabolism of these small muscles markedly synergizes with local changes in metabolic syndrome [48,49]. Therefore, it is also meaningful to consider the action of irisin on these small muscles.

Muscle dysfunction as a factor in metabolic disorders is far more diverse than previously thought. Recently, the interaction between muscle and pancreas has been attracting attention as a predisposing factor for the regulation of insulin secretion. And in the context of the muscle-pancreas interaction, irisin is considered to restore impaired glucose-induced insulin secretion by pancreatic β-cells [20,50]. Considering the importance of muscle in glucose metabolism, developing a blueprint for the regulation of muscle metabolism with myokines will enable the acquisition of further knowledge about the role of the novel myokine in the development and prevention of metabolic disorders [51].

## 4. Effects of Irisin on Muscle Glucose Homeostasis

### 4.1. Skeletal Muscle

Muscle tissue, along with adipose tissue, is considered to be the main target organ for irisin in regulating the homeostasis of glucose [52–55]. In this context, several studies have described that irisin mimicked or reinforced insulin actions in skeletal muscle in vitro and in vivo. That is, the treatment of primary human skeletal muscle cells and the C2C12 myoblast cell line with recombinant irisin for 1 h or longer significantly increased the uptake of glucose [56,57]. Similarly, the overexpression of irisin in C2C12 cells showed a promoting effect on glucose uptake and glycogen accumulation in the cell [57]. In vivo, soleus muscle isolated from irisin-treated (0.1 mg/kg, 4 i.p. injections/week, for 5 weeks) HFD mice contained higher glycogen levels than the control mice by stimulating glucose transporter type 4 (GLUT4) translocation to the skeletal muscle cell membranes, and decreased irisin secretion contributes to muscle insulin resistance [54,57,58]. Furthermore, the irisin-overexpressed C2C12 cells had a significantly higher basal insulin receptor (IR) phosphorylation level than the empty vector-transfected control cells [57]. It has been found that irisin also influences glucose metabolism in skeletal muscle at the level of gene expression. After 6 h of irisin treatment on primary human skeletal muscle cells, the expression of genes that participate in glucose transport and lipid metabolism, such as *GLUT4*, Hexokinase 2 (*HK2*), and peroxisome proliferator-activated receptor alpha (*PPARA*), were upregulated, whereas the expression of genes that relate to glycogenolysis (glycogen phosphorylase; *PYGM*) or gluconeogenesis (phosphoenolpyruvate carboxykinase 1; *PCK1*) was suppressed [59]. These changes in the metabolism of skeletal muscle glucose at various levels were triggered by declines in intracellular and intra-mitochondrial ATP, which led to an increase in the levels of the phosphorylation of AMP-activated protein kinase (AMPK) and the activation of its downstream kinases, such as mitogen-activated protein kinase (MAPK), Erk1/2, and p38 [57,60]. As proof that AMPK is an important factor, a number of papers have shown the importance of the AMPK signaling pathway for the effects of irisin on skeletal muscle glucose metabolism [56,57]. Recombinant irisin augmented the glucose uptake via AMPK activation in differentiated L6 muscle cells [58]. The activation of AMPK was preceded by the induction of reactive oxygen species (ROS) and the activation of p38 MAPK, which was consequential to the translocation of GLUT4 to the outer membranes of these cells [58,61,62]. The treatment of irisin-overexpressed C2C12 cells with compound C, a reversible AMPK inhibitor, suppressed the activity of the IR signaling pathway [57]. Similarly, the enhanced uptake of glucose in the C2C12 cells treated with irisin and cultured in high ambient glucose and PA containing medium were alleviated after the inhibition of the AMPK signaling with AMPKα2 siRNA [62]. The treatment or overexpression of irisin in the C2C12 cell line can attenuate PA-induced insulin tolerance by stimulating the phosphorylation of Akt and Erk [53,57].

Metformin (Met) is a biguanide antihyperglycemic drug that is traditionally used for the management of T2DM [63]. The therapeutic effects of Met are based on a combination of improved peripheral uptake and the utilization of glucose, a decreased hepatic glucose output, a decreased rate of intestinal absorption of carbohydrate, and enhanced insulin sensitivity [64,65]. In skeletal muscle, Met increases glucose uptake through its activation of AMPK [66,67]. Met is also known to promote irisin release from murine skeletal muscle independently of AMPK activation [68], and plasma irisin levels provide clinically relevant information about the effectiveness of Met treatment in T2DM patients [49]. Interaction with irisin in skeletal muscle via AMPK signaling may be one of the mechanisms of action of Met as a therapeutic drug for T2DM.

As mentioned above, it seems plausible to consider that irisin is a regulator of glucose metabolism in skeletal muscle. To put it another way, glucose seems to be a critical factor in regulating irisin synthesis through skeletal muscle. For example, in human studies, myotubes isolated from patients with T2DM expressed higher *FNDC5* levels than those from healthy controls [69]. In these patients, a euglycemic–hyperinsulinemic clamp showed unchanged irisin levels in circulation [70]. Furthermore, the treatment of cultured muscle cells with glucose can reduce *FNDC5* expression significantly [71]. This negative effect of glucose on *FNDC5* expression is more prominent in myotubes isolated from patients with T2DM than in those from healthy controls [72]. These findings suggest that glucose is a critical suppressor of irisin synthesis in skeletal muscle, especially in patients with T2DM [70,71]. It is expected that the details of the mode of involvement of irisin in glucose metabolism in skeletal muscle will be clarified by further research.

### 4.2. Smooth Muscle

There is limited information on the action of irisin on smooth muscle compared to skeletal muscle, and no report regarding the involvement of irisin in smooth muscle glucose metabolism has been published so far. Although not directly related to glucose metabolism, a report demonstrates that platelet-derived growth factor (PDGF)-induced fibrotic phenotype modulation of rat vascular smooth muscle is prevented by irisin through the suppression of the signal transducer and activator of the transcription 3 (STAT3) signaling pathway, and it was suggested that irisin has a function of maintaining a healthy phenotype of smooth muscle cells [72]. It has been reported that the STAT3 pathway induces insulin resistance and the disruption of glucose metabolism in some cells and tissues, such as lung, kidney, and muscle [73–76]. There is also a report showing that intimal hyperplasia can be attenuated by inhibiting the activity of the BB isoform of the PDGF (PDGF-BB)-induced Janus kinase 2 (JAK2)/STAT3 signaling pathway in vascular smooth muscle cells [77]. Taken together, PDGF-STAT3 signaling may contribute to glucose metabolism in smooth muscle cells as well. However, there are reports that the conditional knockout of STAT3 in muscle does not prevent HFD-induced insulin resistance, and STAT3 variants are not associated with obesity or insulin resistance in female twins [78–80]. Further research is needed for details on the relationships among smooth muscle health, PDGF/STAT3 pathway, and glucose homeostasis.

Pioglitazone (PIO), a PPAγ agonist that improves glycemic control in T2DM through its insulin-sensitizing action, was shown to inhibit vascular smooth muscle cell proliferation, and the inhibitory effect was mediated by AMPK activation and/or diminishing of PDGF-induced mechanistic target of rapamycin (mTOR) activity [81]. Membrane-bound PDGF-BB transfer by endothelial cell-derived extracellular vesicles could account for vascular smooth muscle cell resistance to apoptosis under the hyperglycemic environment of patients with T2DM [82]. PDGF-BB specifically induced smooth muscle cell migration and proliferation through PI3K-dependent Akt activation, Erk activation, ROS generation, nuclear factor-κB (NF-kB) and activator protein-1 (AP-1) activation, microRNA (miR)-221 and miR-222 induction, reversion-inducing cysteine-rich protein with kazal motifs (RECK) suppression, and matrix metalloproteinase (MMP2 and 9) activation [83]. According to these studies, it is obvious that various unidentified factors are involved in the action of PDGF. As previously mentioned, information on irisin, smooth muscle, and its glucose metabolism is currently very limited and would be an interesting topic for future research.

### 4.3. Myocardium

It has been reported that, depending on various conditions, rat cardiac muscle may produce more irisin than skeletal muscle in response to an exercise load [84]. This finding showed the possibility that cardiac muscle may be another main source of irisin besides skeletal muscle. This also suggests that myocardium-produced irisin can display endocrine, paracrine, and autocrine functions in cardiac muscle as well as in skeletal muscle.

Among the various myocardial substrates, glucose holds less than 25% of energy generation under ordinary conditions, while fatty acid oxidation generates the majority of energy [85]. However, glucose is unique among myocardial substrates because a small amount of ATP is obtained by substrate-level phosphorylation during glycolysis even in stressful environments, such as hypoxia or ischemia. ATP obtained from glycolysis in the extramitochondrial compartment may be especially critical for the maintenance or restoration of ionic homeostasis. The requirement for glucose to maintain cardiac function becomes more pronounced in the presence of metabolic stress [86]. Therefore, it is important to maintain normal glucose metabolism to sustain the health of the myocardium under stress. Considering the action of irisin on skeletal muscle, it is expected to have a similar effect on glucose metabolism in the myocardium, but so far, no reports have been found on the direct action of irisin in myocardial glucose metabolism. Even in such a situation, there are a few reports that show that irisin has a protective effect on the myocardium in a hyperglycemic environment, with descriptions that may be relevant in no small measure [87,88]. As another example of indirect evidence for effects of irisin on cardiac glucose metabolism, in an in vitro study, 500 μM of PA induced insulin resistance in the H9c2 cardiomyoblast cell line, while co-treatment with 200 ng/mL of irisin reversed it and significantly increased cellular insulin-stimulated glucose consumption by inhibiting autophagy through the PI3K/Akt signaling pathway [89].

Recently, it has been revealed that autophagy plays a pivotal role in diabetes and its cardiac complications [90–92]. Autophagy is a cellular catabolic process, facilitating lysosomal degradation, recycling of intracellular misfolded proteins and injured organelles [93]. It is involved in the maintenance of various physiological responses and plays a dual role in inducing cytoprotection and cell death [94,95]. In the last few years, as one of irisin’s most pleiotropic and favorable properties, irisin’s autophagy regulating function has been attracting attention [96,97]. During the last decade, several studies have described the relationship between autophagy and insulin resistance in cardiac tissue and other organs. However, results and conclusions from these studies have been inconsistent [89,98–100]. The downregulation of autophagy was observed, particularly in autophagy-related 7 (*Atg7*) expression levels in both genetic and dietary models of obesity [101], and in vivo and in vitro suppression of *Atg7* led to impaired insulin signaling. In contrast, suppressed mTOR signaling and augmented autophagy in adipocytes from obese patients with T2DM were described in [102]. Conversely, there is a report showing that autophagy is not involved in the development of insulin resistance in skeletal muscle [103]. In addition, excessive autophagy activation is associated with PA-induced cardiomyocyte insulin resistance [104]. Taken together, these findings may indicate that maintaining normal cellular insulin signaling requires keeping autophagy levels stable. The relationship between autophagy and glucose metabolism is an interesting issue but there is still room for further investigation. As mentioned above, irisin is generally regarded as a regulator of autophagy, and this function of irisin is thought to improve the integrity of cells and tissues [105]. However, currently, there is no clear answer as to how irisin regulates autophagy in the heart or how it attenuates insulin resistance in cardiac muscle. Further innovative reports are needed regarding the relationship between irisin and autophagy.

### 4.4. Effects of Irisin on Mitochondria to Preserve Muscle Glucose Homeostasis

As described briefly above, irisin preserves the mitochondrial transmembrane potential in an AMPK signaling-dependent manner and stimulates mitochondrial biogenesis by upregulating the genetic expression of *Tfam* (mitochondrial transcription factor A), *Ppargc1a*, and *Nrf1* (nuclear respiratory factor 1), as well as the genetic and protein levels of UCP3 and GLUT4 in C2C12 cells [53]. This maintenance of mitochondrial health is associated with the increased resistance of cells to hyperglycemic stress environments [53,58,61].

Mitochondria play a major role in enhancing skeletal function by not only producing ATP to meet energy demands but also by regulating cellular apoptosis and calcium retention [106,107]. The drastic changes in mitochondrial proteome to downregulate mitochondrial metabolic processes have been observed in skeletal muscle in diabetic patients [108,109]. HFD-induced diabetic mice showed mitochondrial dysfunction to inhibit myoblast differentiation [110]. C2C12 myoblasts exposed to high ambient glucose (15 mM) and/or hyper-lipidemic (0.25 mM PA) conditions for 2 h showed increased mitochondrial fragmentation and membrane potential as well as elevated ROS production compared to control cells in normoglycemic (5.6 mM glucose) conditions [111]. Then, autophagy removed damaged mitochondria with metabolic overload to protect the skeletal muscle from insulin resistance in obesity and T2DM [112]. Given these findings, mitochondrial maladaptation to metabolic stress, such as hyperglycemia, can be a critical factor for disturbances of glucose metabolism in skeletal muscle. However, there is also a report showing that mitochondria are functionally intact in insulin-resistant skeletal muscle from a T2DM non-obese rat model [113], so further verification is necessary on this matter. Exercise is an effective nonpharmacological remedy that induces beneficial mitochondrial adaptations, increasing mitochondrial quality and content [114]. The exercise-induced mitochondrial adaptations in skeletal muscle act on PGC1α, which activates the downstream factor FNDC5 in the skeletal muscle cells [115]. This intriguing relationship between FNDC5/irisin and mitochondrial genes and proteins that regulate mitochondrial function has recently been reported [116,117].

### 4.5. The Effects of Irisin on Systemic Glucose Homeostasis

#### 4.5.1. Interactions of Irisin and Other Hormones

The effects of irisin on skeletal muscle and the interaction of irisin with other hormones were well described in a previously published review [60]. That is, irisin induced a significant increase in levels of betatrophin (also known as angiopoietin-like protein 8) in obese mice [118]. In mice, betatrophin is produced by the liver, WAT, and brown adipose tissue (BAT), while in humans, the liver is the major producing organ [119]. Betatrophin affects glucose homeostasis and lipid metabolism [120]. Accordingly, a PGC1α–irisin–betatrophin pathway has been expected to regulate glucose homeostasis. According to this theory, exercise-induced PGC1α stimulates FNDC5 expression and consequently increases irisin release from muscle cells, and then irisin acts on muscle in a paracrine or autocrine manner to reduce insulin resistance directly and/or indirectly through betatrophin. However, some studies could not reproduce these previous results and the role of betatrophin in glucose homeostasis, and even the existence of such an axis, remains controversial [121].

Leptin participates in glucose homeostasis with irisin. Leptin mediates stimulation in myotubes, the downregulation of irisin secretion, and the expression of FNDC5 in subcutaneous adipose tissue (SAT) [121]. Leptin can also induce irisin-dependent myogenesis and inhibit the browning of adipocytes by downregulating UCP1 [122]. Interactions between other adipokines, such as adiponectin or resistin and irisin, have also been described. For example, a positive association between serum levels of irisin and adiponectin has been described in obese patients [123], while a negative relationship of irisin with resistin has been found in exercise training [124]. Of note, studies associating irisin concentrations with adipokines are still scarcely described and contradictory. There are descriptions that there is both a correlation or no correlation between the expression levels of irisin and leptin [125,126]. A cohort study on children has reported no correlation between the levels of irisin and resistin [127]. Several studies have described the interaction between leptin and irisin. Leptin increased the expression of *FNDC5* in the skeletal muscle of mice while decreasing *FNDC5* expression in SAT via the downregulation of *PGC1α*. Co-treatment with leptin and irisin downregulated irisin-induced fat browning of subcutaneous adipocytes [128]. Thus, further characterization of the relation between irisin and adipokines, a potential factor involved in cardiometabolic risk, is needed in the future. Finally, so far, there is not much available information on the relationship between irisin and other hormones involved in glucose metabolism, such as adrenaline, cortisol, growth hormone (GH), and incretins. Diurnal fluctuations are observed in the blood level of irisin, meaning that the possibility that irisin and cortisol/growth hormone are mutually regulated cannot be ruled out, as these hormones follow a specific circadian circulating pattern [129]. Furthermore, serum levels of irisin in individuals with a various range of body mass index (BMI), including patients with anorexia nervosa or those with obesity, show no relation to levels of cortisol, TSH, C-reactive protein, or ghrelin [130]. However, as mentioned above, a possible relation of irisin with these hormones has not been described in detail yet, and it is premature to carry out a detailed discussion on that.

Only a few studies have reported the role of irisin in insulin signaling. In these reports, in vitro C2C12 myofibroblasts treated with PA have increased insulin resistance via the suppression of Akt and/or MAPK (Erk1/2 and p38) phosphorylation, and this suppression was partially reversed by irisin, indicating a protective effect of irisin on insulin signaling in muscle [57,58]. Moreover, several studies described a direct correlation between fasting levels of irisin and insulin but not between postprandial levels of them [130–132]. Conversely, insulin did not alter irisin levels in patients with T2DM and obesity in a euglycemic–hyperinsulinemic clamp [107]. Due to its modalities of secretion and its pancreatic and extra-pancreatic effects, irisin could be considered an incretin-like hormone, with an action similar to that of glucagon-like peptide-1 (GLP-1), which retains substantial insulinotropic activity in diabetic patients [133]. This similarity between irisin and incretin has been discussed but not yet established. Future studies should focus on irisin’s insulinotropic effect and on any possible interactions between irisin and insulin that might affect glucose metabolism.

#### 4.5.2. Interventional Animal Studies

In the very first report introducing irisin, BALB/c mice fed with an HFD for 20 weeks were injected intravenously with FNDC5-expressing adenoviral particles [17]. After 10 days, these mice had similar body weights to the control mice, however, the glucose levels and fasting levels of insulin after intraperitoneal glucose infusion were significantly reduced (~50%), suggesting that irisin can attenuate systemic insulin resistance. Regarding the autocrine physiological effects of irisin on muscles, the in vivo treatment of mice with irisin resulted in an increase in muscle mass and strength [134]. In the study, 5-week-old C57BL/6J mice were injected twice weekly with 2.5 μg/g body weight of irisin intraperitoneally (IP) for 4 weeks and changes in weight and the cross-sectional area (CSA) of muscles were evaluated (quad, M. biceps femoris, M. tibialis anterior, and M. extensor digitorum longus) along with some biochemical/histochemical markers. With these data, the authors of the paper proposed that irisin injection leads to an increase in the activation of satellite cells and reduces protein degradation by the downregulation of atrogin-1 and muscle ring-finger protein-1 (MuRF-1), resulting in a partial rescue of muscular atrophy. As an investigation for the potential autocrine role of irisin on skeletal muscle glucose metabolism, Yang et al. showed that HFD-induced diabetic C57BL/6 mice developed muscular impairment of insulin signaling, and in combination with the in vitro data, proposed that extrinsic irisin reverses the insulin resistance of the myocytes [55]. Moreover, Farrash et al. reported that the electrotransfer of *FNDC5*-harboring vectors to rat hindlimb muscle (M. tibialis cranialis) resulted in the increase of muscle glycogen, along with enhanced glycogen synthase 1 (GS1) gene expression [135]. In addition, GLUT4 protein tended to increase in the muscle [135]. However, glucose uptake by the muscle was unchanged, suggesting that short-term in vivo effects of irisin on muscle glucose uptake were not defined in the study.

#### 4.5.3. Human Studies

A number of clinical studies regarding the relation between irisin and systemic glucose metabolism have been published. For example, Park et al. reported that serum irisin levels are associated with an increased risk of metabolic syndrome in humans, indicating either increased irisin secretion by adipose/muscle tissue or a compensatory increase of irisin to overcome an underlying irisin resistance [136], which is similar to the well-documented leptin resistance [137]. Irisin resistance is generally defined as the inability of endogenous or exogenous irisin to promote the expected beneficial metabolic outcomes, such as stimulation of energy expenditure, due to multiple molecular, neural, environmental, and behavioral mechanisms. María et al. proposed that in individuals with obesity, *FNDC5* expression in muscle was significantly decreased in association with T2DM, and *FNDC5* expression in muscle was significantly associated with *FNDC5* and *UCP1* expression in visceral adipose tissue [133]. In most clinical studies, irisin levels of patients with pre-diabetes or T2DM have been reported to be lower than the controls [134,138,139]. The factor that is responsible for the low secretion of irisin in T2DM has not yet been identified, although some studies have suggested that chronic hyperglycemia and hyperlipidemia are possible causes [37,70]. Accordingly, the levels of irisin in the blood could be an important factor in the changes observed in the health and disease of metabolism [140]. Taken together, although there seems to be no doubt that irisin is associated with insulin resistance, there is no consensus on the link between irisin and metabolic syndrome.

Furthermore, there is no publication for human study about the mechanism by which the effect of irisin on muscle glucose metabolism leads to systemic obesity and insulin resistance. This lack of literature is probably due to the difficulty in the evaluation of glucose metabolism in the living body. Larger prospective studies with the innovation in research technology are therefore needed to clarify these issues.

A list of the animal and human study papers to note is summarized in Table 1 [16,20,34,37,50,54,70,134–136,138,139,141–145].

### 4.6. Applicability of Irisin in the Treatment of Diabetic Complications

T2DM, especially with its major complications (neuropathy, retinopathy, and nephropathy) is known to be associated with the increased risk of loss of mobility and strength that is frequently associated with disease control. Sarcopenia, a comorbid symptom of T2DM, is a loss of muscle mass associated with a loss of strength and/or performance, resulting in worse morbidity and quality of life in patients [146]. Currently, practical treatments are limited to indirect means, such as dietary prophylaxis and exercise therapy. With the increasing prevalence of sarcopenia in T2DM, there is a need for new interventions that effectively counter the loss of skeletal muscle mass. Considering the direct effects of irisin to preserve the health of skeletal muscle, irisin may also have potential as a treatment for sarcopenia. Furthermore, diabetic foot ulceration (DFU) occurs in up to one-quarter of people with T2DM and is one of the most common causes of lower limb amputation [147]. Wounds of diabetic patients usually show abnormal slow healing, and this delayed healing is thought to be due to a combination of factors, including macrovascular and microvascular disease [148]. Angiogenesis, the formation of new blood vessels from pre-existing vessels, is a crucial process for wound healing and is seriously damaged in diabetic wounds [149]. Irisin improved cardiac function and reduced the infarct area in post-myocardial infarct mice hearts, and this therapeutic effect was associated with its pro-angiogenic effects [150]. Based on these findings, it is possible that irisin may also have a therapeutic effect on DFU by a mechanism other than the normalization of muscle glucose metabolism. This effect was partly due to the reduction of oxidative stress (due to a decrease in intracellular ROS levels and an increase in the total antioxidant capacity) by suppressing inflammatory markers such as NF-κB, cyclooxygenase 2, p38 MAPK, tumor necrosis factor (TNF), and IL-6 [151,152]. Taken together, irisin not only keeps muscle glucose metabolism healthy in a hyperglycemic and high-lipid environment but also has the effects of maintaining the health of tissue oxidative/antioxidant balance and suppressing inflammation, so it can be a potential therapy not only for T2DM but also for many of its complications.

## 5. Conclusions

**Figure 3.**
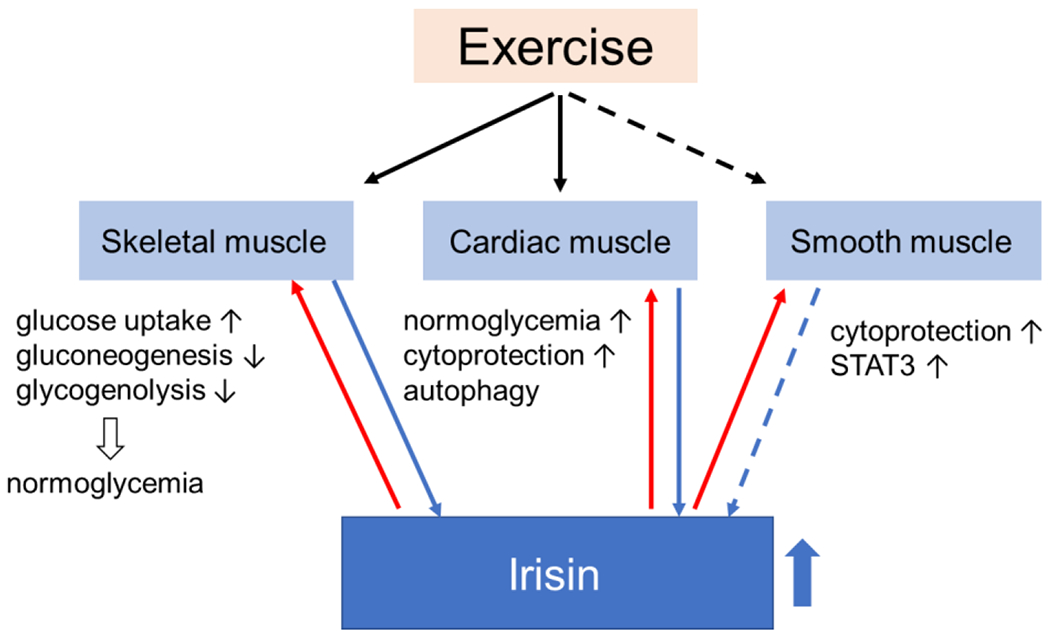
Irisin is primarily secreted by skeletal and cardiac muscle (and maybe by smooth muscle) during exercise (blue arrows). Irisin returns to muscles via blood or in an autocrine manner (red arrows), leading to changes in their handling of glucose homeostasis. The effects of irisin on muscles favor states of normoglycemia. Black arrows pointing up indicate promotion and black arrows pointing down indicate suppression.

Muscle, one of the major targets of insulin, is one of the first tissues to develop insulin resistance in a state of general obesity, diabetes, and other forms of disorders of glucose metabolism. Considering the function of muscle as an endocrine organ that secretes a variety of myokines involved in maintaining homeostasis of glucose metabolism in response to nutritional status and exercise, it is reasonable to imagine that the development of insulin resistance in muscle has a great effect on its function as a secretome or vice versa. Since muscle is also the major tissue where insulin stimulates glucose uptake and removes excess glucose from the blood, it plays a central role in glucose metabolism throughout the body, so that changes in the muscular secretome may have an impact not only on the local muscle but also on the systemic glucose homeostasis. Irisin, which has been known to be involved in the regulation of energy expenditure, seems to be a strong candidate for the treatment of metabolic disorders. In fact, its potential as a therapy has been suggested by numerous in vivo and in vitro experiments. Through functions in muscle, irisin contributes to normoglycemia (Figure 3).

The elucidation of irisin’s physiology involved in the maintenance of muscle and systemic glucose homeostasis and understanding their mechanisms of action is critical in developing treatments for metabolic diseases, such as obesity and T2DM, by pharmacologically mimicking the effects of exercise. Based on the current knowledge, trials to evaluate the usefulness of irisin as a therapeutic agent in humans appear to be premature. That is, many reports have not reproduced the previous findings, partly because non-physiological levels of irisin were used in these studies, as many of them were done before it became possible to accurately measure the blood levels of irisin. Furthermore, inconsistencies in the data highlight the necessity for better design for both basic and clinical studies. In recent years, the accuracy of the irisin assay has improved, and the accumulation of irisin’s physiological information, such as concentration in circulation, has also progressed. Thus, it is expected that the accuracy and consistency of irisin research will be improved in the future.

## Figures and Tables

**Table 1.** List of animal/human studies for irisin and glucose metabolism.

		Author	Year	PMID*	Ref#**
**1. Animal studies**					
1.	A PGC1-α-dependent myokine that drives brown-fat-like development of white fat and thermogenesis	Boström et al.	2012	22237023	[16]
2.	The myokine irisin is released in response to saturated fatty acids and promotes pancreatic β-cell survival and insulin secretion	Natalicchio, et al.	2017	28724742	[20]
3.	FNDC5 overexpression and irisin ameliorate glucose/lipid metabolic derangements and enhance lipolysis in obesity	Xiong, et al.	2015	26111885	[34]
4.	Irisin ameliorates glucolipotoxicity-associated β-cell dysfunction and apoptosis via AMPK signaling and anti-inflammatory actions	Zhang, et al.	2018	30466091	[50]
5.	Decreased irisin secretion contributes to muscle insulin resistance in high fat diet mice	Yang, et al.	2015	26261526	[54]
6.	Irisin is a pro-myogenic factor that induces skeletal muscle hypertrophy and rescues denervation-induced atrophy	Maisha Reza, et al.	2017	29062100	[134]
7.	Impacts of rat hindlimb Fndc5/irisin overexpression on muscle and adipose tissue metabolism	Farrash, et al.	2020	32369414	[135]
8.	Effects of irisin and exercise on metabolic parameters and reproductive hormone levels in high-fat diet-induced obese female mice	Bastu, et al.	2018	28594316	[141]
**2. Human studies**					
1.	Effects of obesity, diabetes and exercise on Fndc5 gene expression and irisin release in human skeletal muscle and adipose tissue: in vivo and in vitro studies	Kurdiova, et al.	2014	24297848	[37]
2.	Irisin is expressed and produced by human muscle and adipose tissue in association with obesity and insulin resistance	Moreno-Navarrete, et al.	2013	23436919	[70]
3.	Circulating irisin in relation to insulin resistance and the metabolic syndrome	Park, et al.	2013	24057291	[136]
4.	Serum irisin levels and clinical implication in elderly patients with type 2 diabetes mellitus	Xuan, et al.	2020	32849950	[138]
5.	Serum irisin levels, endothelial dysfunction, and inflammation in pediatric patients with type 2 diabetes mellitus and metabolic syndrome	Huerta-Delgado, et al.	2020	32964051	[139]
6.	Association between circulating irisin levels and the promotion of insulin resistance during the weight maintenance period after a dietary weight-lowering program in obese patients	Crujeiras, et al.	2014	24439241	[142]
7.	Irisin levels before and after physical activity among school-age children with different BMI: a direct relation with leptin	Palacios-González, et al.	2015	25820255	[143]
8.	Effects of body weight reduction on serum irisin and metabolic parameters in obese subjects	Fukushima, et al.	2016	27766246	[144]
9.	Effect of long-term moderate physical exercise on irisin between normal weight and obese men	Rashid, et al.	2020	32952453	[145]

* PubMed ID;

** Citation number in the text.

## Data Availability

Not applicable.
